# Mast Cell Activation Protects Cornea by Promoting Neutrophil Infiltration via Stimulating ICAM-1 and Vascular Dilation in Fungal Keratitis

**DOI:** 10.1038/s41598-018-26798-3

**Published:** 2018-05-30

**Authors:** Yanting Xie, Hongmin Zhang, Susu Liu, Guoming Chen, Siyu He, Zhijie Li, Liya Wang

**Affiliations:** 1 The First Affiliated Hospital of Zhengzhou University, Zhengzhou, 450003 People’s Republic of China; 2 Henan Eye Institute, Henan Eye Hospital, Henan Provincial People’s Hospital, Department of Ophthalmology, Zhengzhou, 450003 People’s Republic of China

## Abstract

The role of mast cells (MCs) in fungal infection is largely unknown. This study was to explore a protective role and mechanism of MCs in fungal keratitis. Experimental fungal keratitis (FK) mouse model was developed. Mice untreated (UT) or receiving corneal wound without fungal infection (Mock) were used as controls. Large number of connective tissue MCs was found in normal mice. MC activation with degranulation was largely observed, and the percentage of degranulated/total cells was high in FK. Dilated limbal vasculature with increased permeability, as well as largely infiltrated neutrophils with stimulated ICAM-1 protein levels were observed in corneas of FK mice, when compared with Mock and UT mice. Interestingly, pretreatment with cromolyn sodium (Block) significantly blocked MC degranulation, dramatically suppressed vascular dilation and permeability, and markedly reduced neutrophil infiltration with lower ICAM-1 levels in FK mice at 6–24 hours. Furthermore, the Block mice manifested prolonged disease course, increased pathological damage, and vigorous fungus growth, with much higher corneal perforation rate than FK mice at 72 h. These findings reveal a novel phenomenon that MCs play a vital role in protecting cornea against fungal infection through degranulation that promotes neutrophil infiltration via stimulating ICAM-1 production and limbal vascular dilation and permeability.

## Introduction

Fungal keratitis (FK), also known as keratomycosis or mycotic keratitis, is an infection caused by opportunistic pathogenic fungi. In the developing countries, it occurs mainly among male populations at low levels of education, who engage in agricultural activities, and is frequently accompanied by a history of plant trauma. Most patients eventually require surgery (59.45%), at a low level of pharmaceutical cure rate (40.55%)^1^. In the large sample of clinical epidemiology studies, *Fusarium*^2–8^, *Aspergillus*^9^, and *Candida albicans* were the main pathogens of FK, and *Fusarium* manifests with more serious symptoms and poor efficacy of treatment^10^.

In fungal diseases, different types of immune cells are involved in the antifungal process with distinct mechanisms. Infiltrated inflammatory cells were mainly neutrophils followed by macrophages in corneas of patients and mice^11^. Neutrophils are attracted by chemokines in the affected area via neutrophil surface integrins. Then these immune cells subsequently transmigrate from blood vessels of limbus to the site of infection. Neutrophils can distinguish the site of the micro-organisms to initiate endocytosis and release extracellular traps, and final kill micro-organisms^12^.

Mast cells (MCs) are bone marrow progenitor–derived immune cells that mature in tissues affected by the local microenvironment. MCs are mainly distributed in the transitional areas between the body and outside. MCs can participate in numerous biological processes, including infection control, wound healing, inflammation and immune tolerance. In infectious diseases, MCs play a critical role by releasing previously stored particulate materials as well as new synthetic substances in vascular permeability and angiogenesis^13–15^, fibroblast proliferation, and scarring formation. MCs also induce the expression of ICAM-1 in endothelial cells through the release of cytokines to promote neutrophil chemotaxis^16^ and perform antimicrobial actions by secreting antimicrobial peptides defensins^17^. Studies on corneas infected by virus in MC-knockout mice manifest increased virus load and leukocyte infiltrations, but decreased corneal transparency^18^. When MCs encountered *Candida albicans*, they can digest spores by endocytosis^19^. After toe pads were inoculated with the fungus, MC activation-associated proteins can be detected in the serum of guinea pigs^20^. However, no direct data demonstrated the role of MCs or their anti-fungal mechanism in fungal keratitis.

To investigate how MCs influence corneal fungal infection, we established the mouse model of fungal keratitis with *Fusarium Solani* to explore possible functions and mechanisms of MCs during fungal infection in the cornea. We found that MCs activation initiated by fungi infection contributed to the neutrophil infiltration by influencing the expression of ICAM-1 to the infected cornea and result in protection in FK.

## Results

### Connective tissue mast cells were observed to be located in the corneal limbus

**Figure 1:**
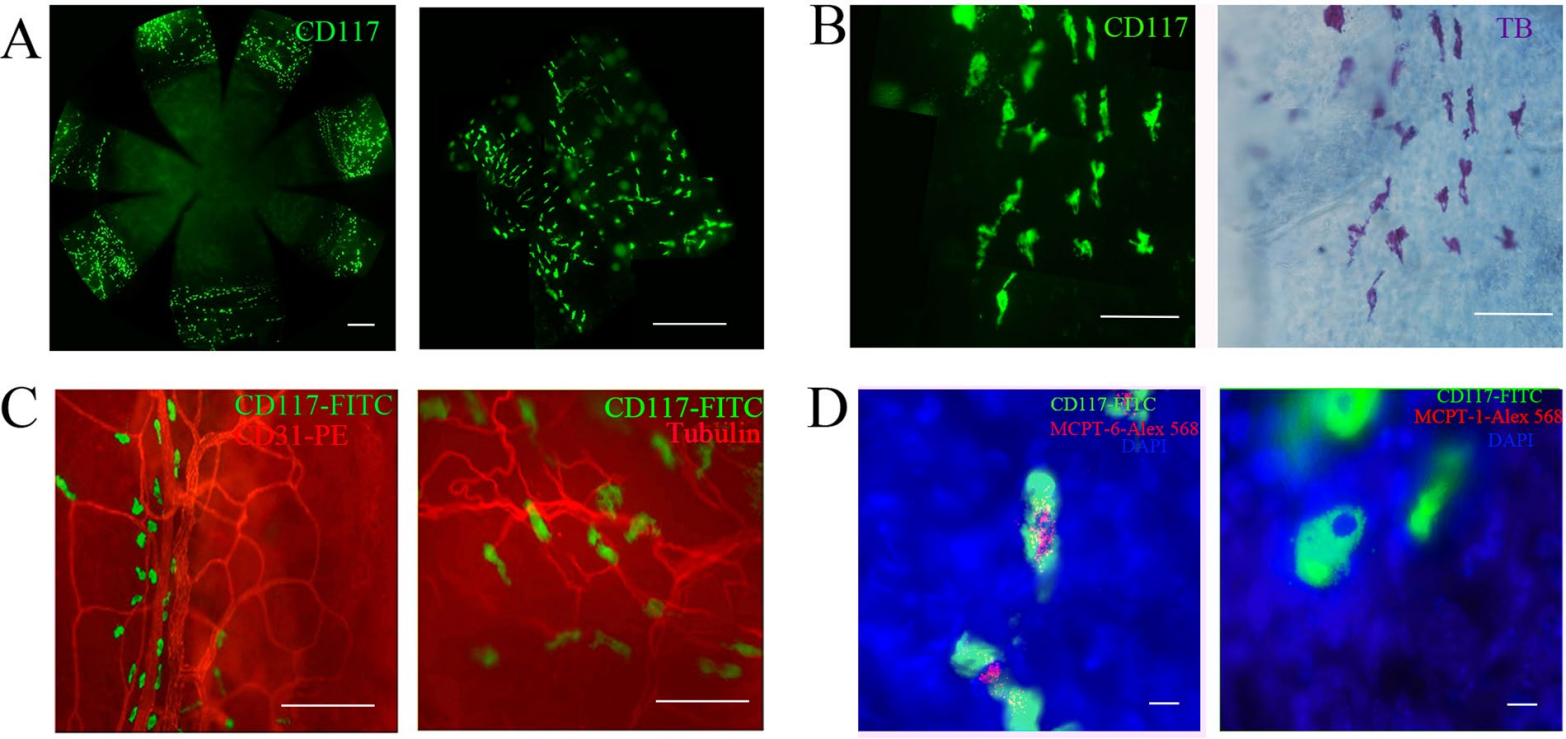
Connective tissue mast cells were verified in cornea. (**A**) The corneas of normal mice were cut into eight pieces after immunofluorescent staining. In the whole cornea, there were no CD117-positive cells in central, paracentral and peripheral cornea (left). CD117-positive cells were present on the corneal limbus and conjunctiva (right). Scale bar for 500 μm. (**B**) The fluorescent images (×20) captured from one piece of the cornea merged into a single image (left). Photos (×20) of the same area were taken under a light microscope after toluidine blue O (TB) staining (right), Scale bar for 100 μm. (**C**) MCs (green) were located around the blood vessel (left, red) and nerve (right, red) in the corneal limbus. Scale bar for 100 μm. (**D**) MCPT-6 positive (left, red, 100×) and MCPT-1 negative (right, 100×) Cells were verified CTMC in cornea. Green indicated the MCs, and blue indicated nuclei. Scale bar for 10 μm.

MCs are distributed in the ocular surface^21–23^. To reveal the characteristics of these cells, we used classic staining methods—toluidine blue staining combining the whole-mount technique to verify the distribution of MCs in the cornea and conjunctiva. Consistent with previous studies, we found the presence of a large number of MCs in the conjunctiva (Fig. 1A) and limbus. These cells were toluidine blue and CD117 positive (Fig. 1B), which was a constructive expressed molecular on the MC during maturation. MCs also located near to the blood vessels and nerves (Fig. 1C).

MCs have been known to consist of at least two types, connective tissue mast cells (CTMCs) and mucosal mast cells (MMCs) based on to their locations and enzymes. Some researchers show both types of cells may co-exist in some areas. Studies show that mouse MC protease (mMCPT)-6 was specifically expressed in CTMC while mMCPT-1 existed only in MMCs. To determine the MC subtypes in the limbus, the corneal whole mounting and immunofluorescence staining technique was applied. Our results found that the MCs in limbus were mMCPT-6 positive but mMCPT-1 negative cells (Fig. 1D), indicating they were CTMCs.

### MCs were largely activated in fungal keratitis

**Figure 2:**
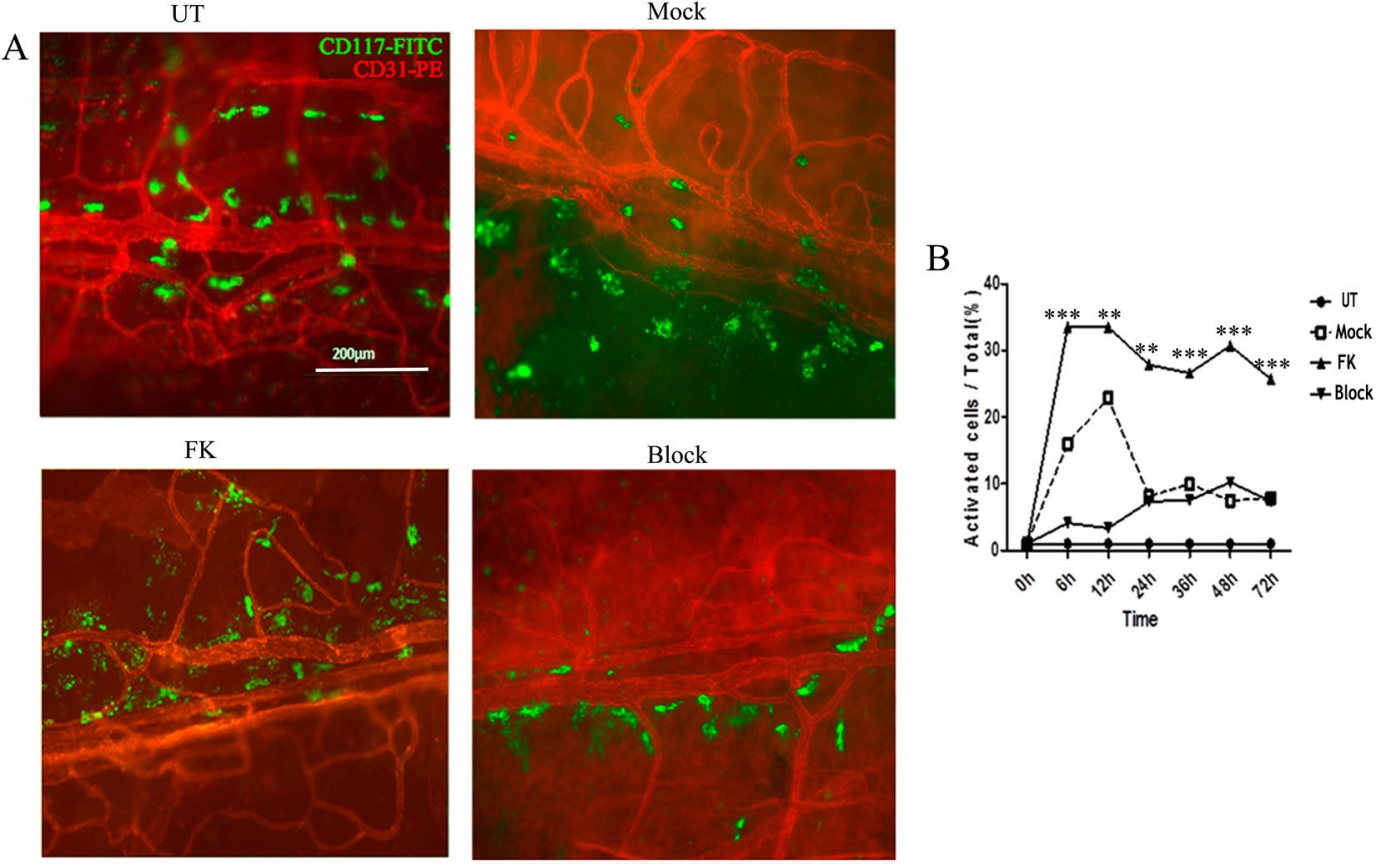
Mast cells were activated by fungal irritation. (**A**) Corneal whole mounts were used for the MC activation morphological analysis. The UT cornea was compared to the Mock group, FK group and Block group after 24 h of stimulation with the fungi or trauma. Cromolyn sodium suppresses the degranulation of mast cells at 0, 6, 12, 24 h. (**B**) The ratio of activated/total MCs per cornea (5–6 eyes per group) in the UT group, mock group, and FK group at different times were analyzed. Scale bar for 200 μm. *P < 0.05, **P < 0.01, ***P < 0.001.

MC cytoplasm contains a large number of particles. When MCs are irritated by parasites, bacteria, and other pathogens, granules wrapped in cytoplasm are released into the matrix of surrounding tissue^24^. In order to observe whether the fungal infection trigger MC degranulation in the cornea, we established FK models to observe the presentation of activated (degranulated) MC in corneal limbus. We found that a large number of particles were around the MCs after 6 hours of corneal irritation by fungal infection or trauma (Fig. 2A). The number of MCs was 645 ± 26 cells per cornea in normal mice, and no significant change of the MC number was observed in Mock, FK and Block group. The percentage of degranulated over total MCs in FK-infected corneas was 37.87%, 37.27%, 27.88%, 26.63%, 30.71%, 25.75% at 6 h, 12 h, 24 h, 36 h, 48 h and 72 h, respectively, which were significantly higher than that in Mock and Block (Fig. 2B).

### Mast cell degranulation stimulated vessels dilation in corneal limbus

The vascular diameter controls the speed of blood flow and numbers of immune cell infiltration. In order to determine the effect of MCs on blood vessel, we utilized cromolyn sodium, immunofluorescence staining, and Evans blue (EB) to detect changes in the vessel diameter and vessel permeability in the process of FK.

**Figure 3:**
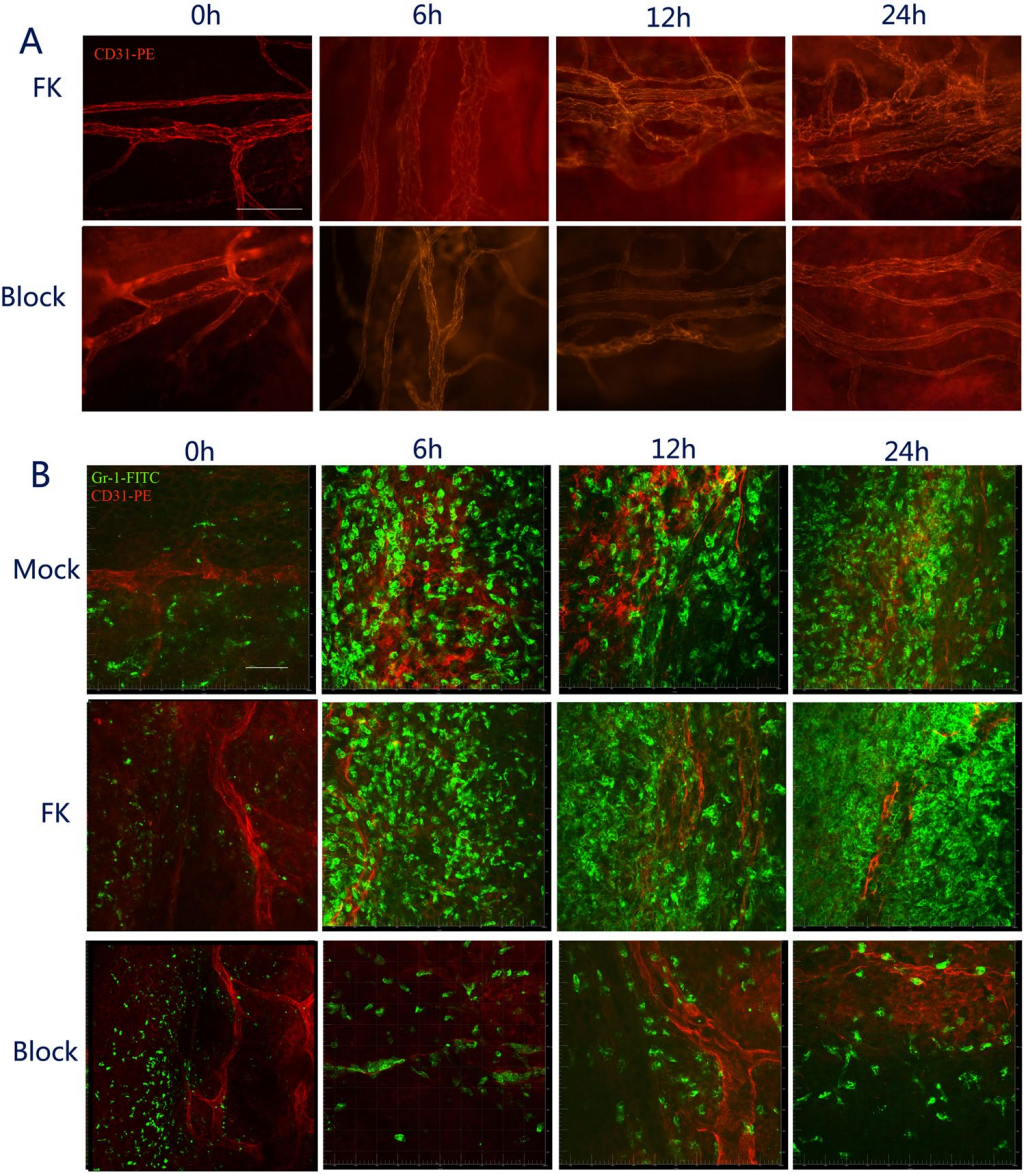
Vessel dilation and neutrophils infiltration were suppressed by blocking of mast cell degranulation. (**A**) After fungi or trauma irritation, The CD31-PE labeled vessels (red) in the limb were also captured using a fluorescence microscope in the FK group (top) and Block group (bottom) at 0 h, 6 h, 12 h, and 24 h. Scale bar for 100 μm. (**B**) Gr-1-FITC labeled neutrophils were also captured by confocal laser scanning microscopy in the Mock group (top), FK group (middle), and Block group (bottom) at 0 h, 6 h, 12 h, and 24 h. Scale bar for 40 μm.

**Figure 4:**
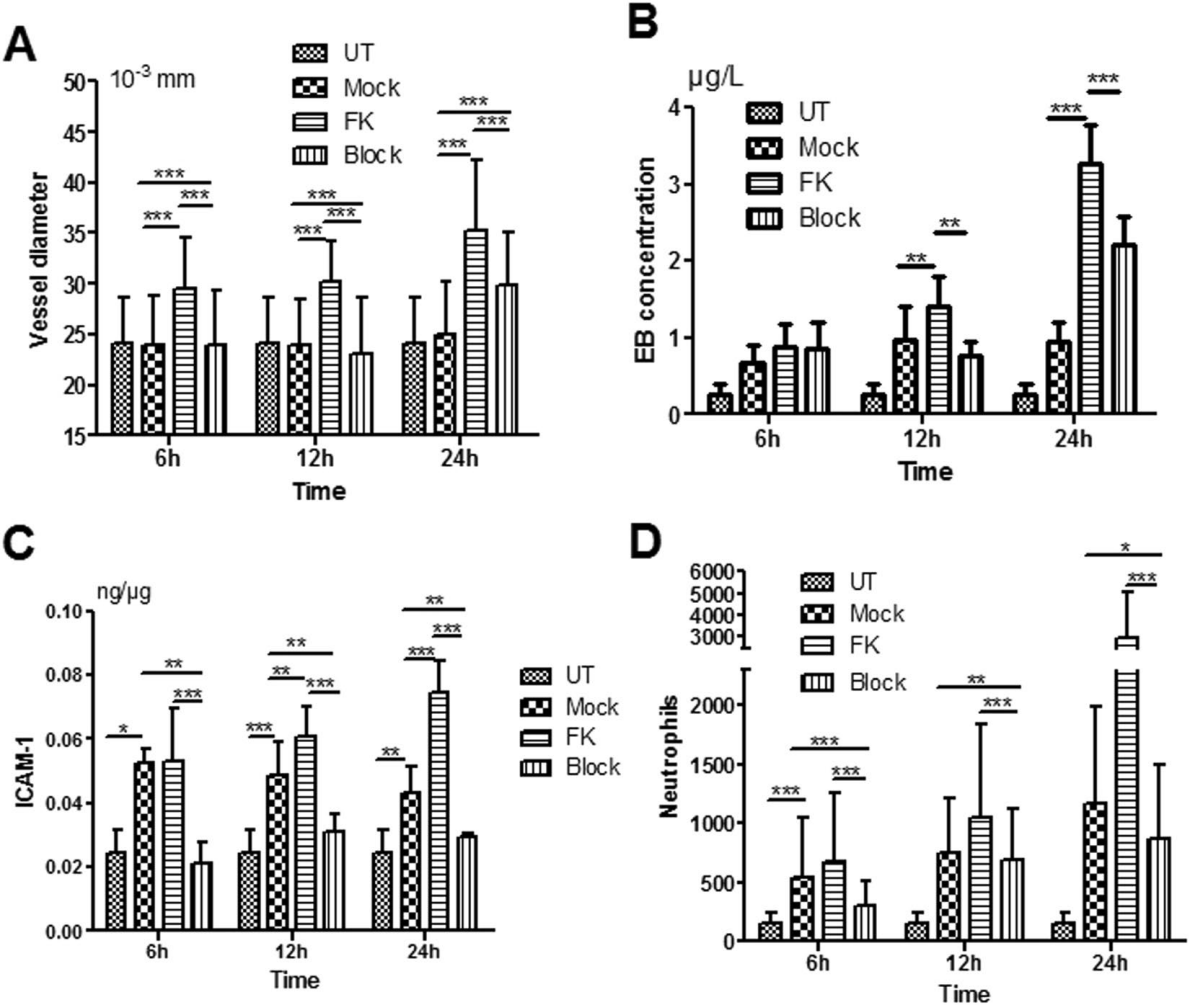
Blocking of mast cell degranulation depressed vessel changes, ICAM-1 expression and neutrophils infiltration. (**A**) The vessel diameters of the Block group was compared to the UT group, Mock group, and FK group at 6 h, 12 h, and 24 h after the fungi infection (n = 5 or 6 per group). (**B**) The blood vessel permeability of the corneal limbus were examined with EB concentration (n = 5 or 6, per group). (**C**) ICAM-1 concentration in the Block group was compared to the UT group, Mock group, and FK group at 6 h, 12 h, and 24 h after fungi infection (n = 6 per group). (**D**) The number of infiltrating neutrophils (Gr-1 + cells) were analyzed at 6 h, 12 h and 24 h (n = 5 per group). *P < 0.05, **P < 0.01, ***P < 0.001. The data are representative of two independent experiments.

The vessel diameter was 24.06 ± 4.63 μm in normal corneas (Figs 3A and 4A), and it was 24.21 ± 4.79, 24.38 ± 4.46 and 25.67 ± 5.15 μm at 6 h (*P* = 0.830), 12 h (*P* = 1.000) and 24 h (*P* = 0.250), respectively, in the Mock group, which was not statistically different. But it considerably widened to 29.53 ± 5.23, 30.14 ± 4.36, 34.09 ± 6.29 μm at 6 h (*P* < 0.01), 12 h (*P* < 0.01) and 24 h (*P* < 0.001) respectively, in FK mice. When MC activation was blocked, the vessel diameter remained at low levels with 23.96 ± 5.34 μm at 6 h, 23.02 ± 5.67 μm at 12 h, which was as same as the Mock group at 6 h (*P* = 0.706), 12 h (*P* = 0.108), but lower than that of the FK group (6 h, *P* < 0.001; 12 h, *P* < 0.001). Although the vessel diameter of the Block group rose to 31.06 ± 5.97 μm at 24 h, which higher than that of the Mock group (*P* < 0.001), it was still lower than FK group (*P* = 0.028) (Figs 3A and 4A).

The EB concentration used to analyze vascular permeability (Fig. 4B) was at a low level of 0.26 ± 0.03 μg/L in normal corneas. The EB concentration of the Mock group was considerably increased to 0.80 ± 0.27, 0.90 ± 0.37, 0.96 ± 0.25 μg/L at 6 h, 12 h, and 24 h respectively, higher than that of normal corneas (6 h, *P* < 0.001; 12 h, *P* = 0.001; 24 h, *P* < 0.001). The EB concentration of the FK group sustainably increased to 0.95 ± 0.26, 1.45 ± 0.39, 3.54 ± 0.17 μg/L at 6 h, 12 h and 24 h respectively in FK group, and higher than that the Mock group at 12 h and 24 h (12 h, *P* = 0.001; 24 h, *P* < 0.001). When MC activation were blocked, the EB concentration were decreased to 0.95 ± 0.20, 2.47 ± 0.35 μg/L at 12 h and 24 h, lower than the FK group (12 h, *P* = 0.009; 24 h, *P* < 0.001).

### Mast cell degranulation increased ICAM-1 expression

In addition to vascular changes, ICAM-1 is crucial for neutrophil migration into the sites of infection through the vascular wall^25,26^. An enzyme-linked immune-sorbent assay was used to investigate whether ICAM-1 protein expression changed by MC activation.

Intercellular adhesion molecule varied following the vascular dilation and increased permeability during inflammation in FK model, Mock group, and Block group (Fig. 4C). We confirmed that ICAM-1 expression is at a low level (27.27 × 10^−3^ ± 9.24 × 10^−3^ ng/μg) in normal corneas, MC activation irritated by fungi triggered the expression of ICAM-1 to 61.15 × 10^−3^ ± 9.61 × 10^−3^, 63.53 × 10^−3^ ± 11.18 × 10^−3^, 69.28 × 10^−3^ ± 9.61 × 10^−3^ ng/μg at 6 h, 12 h and 24 h, respectively (Fig. 4C). After MCs activation was pharmacologically blocked, ICAM-1 expression decreased to 21.01 × 10^−3^ ± 6.51 × 10^−3^ ng/μg at 6 h, 31.04 × 10^−3^ ± 5.57 × 10^−3^ ng/μg at 12 h and 29.40 × 10^−3^ ± 1.22 × 10^−3^ ng/μg at 24 h, which was similar to the UT mice (6 h, *P* = 0.499;12 h, *P* = 0.445; 24 h, *P* = 0.646) and significantly lower than the FK group (all *P* value < 0.001).

### Mast cell degranulation promoted neutrophil infiltration

Neutrophils are the earliest and the most innate immune cells infiltrating to the infected area during earlier stage of fungal keratitis^16^. They play a crucial role in killing fungi during infection^27^. To determine the role of MCs in the chemotaxis of neutrophils, we used cromolyn sodium, the mouse model of FK, and immunofluorescence staining to observe chemotaxis of neutrophil to the central cornea.

We found immune cell infiltration at the early stage of fungal keratitis. In wild type mice, neutrophils were only distributed in the corneal limbus with a density of 248 ± 68 cells per cornea (Figs 3B and 4D). Neutrophil infiltration increased to 1085 ± 57 cells at 6 h in the Mock group, and also increased slightly to 1216 ± 298, 1987 ± 340 cells at 12 h and 24 h, respectively. Neutrophil infiltration in the FK group significantly increased to 1258 ± 98 cells, in contrast to the Mock group (*P* = 0.023) at 6 h; it was increased to 1847 ± 258 cells at 12 h (*P* = 0.015) and 5046 ± 258 cells at 24 h (*P* < 0.001), significantly higher than that of Mock group. When MC activation was blocked, the number of neutrophils was decreased to 511 ± 92 cells at 6 h, lower than the Mock group (*P* < 0.001) and FK group (*P* < 0.001), but similar to normal corneas (*P* = 0.094, Figs 3B and 4D). After 12 h and 24 h stimulation by fungi, the neutrophil infiltration in the Block group showed a very small increase, but was still significantly lower than the trauma group (*P* = 0.040, *P* = 0.011) and FK group *(P* < 0.001, *P* < 0.001) (Figs 3B and 4D).

### Mast cell activation was related to clinical and pathological process of fungal keratitis

**Figure 5:**
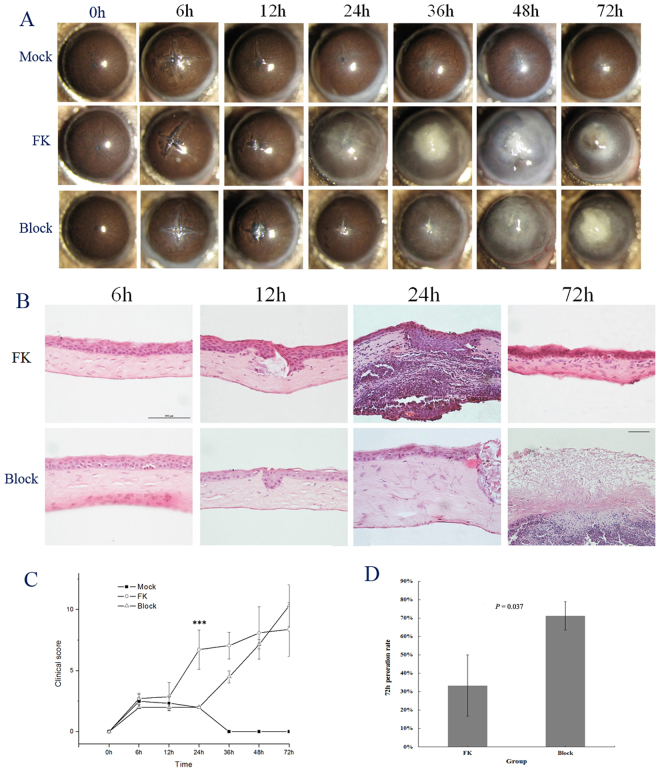
Mast cells activation decreased peroration rates. Photographs showing changes in the corneas were taken under slip-lamp microscopy (**A)** at 0 h, 6 h, 12 h, 24 h, 36 h, 48 h, 72 h after fungi or/and trauma irritation. HE staining were performed to observe the general pathological changes at different time points (**B**). Clinical scores (**C**) were assessed under slit-lamp microscopy at 6 h, 12 h, 24 h, 36 h, 48 h, and 72 h after the hypha treatment. The peroration rates (**D)** of the Block group and FK group were compared (6 per group, 4 batches, mean and s.d,) at 72 h. *P < 0.05, **P < 0.01, ***P < 0.001.

To investigate the effects of MC activation on the clinical process and pathological change of FK, we treated animals with cromolyn sodium and observed the corneal changes with a slit lamp microscope and pathological staining. Before (0 h) and after (6 h, 12 h, 24 h, 36 h, 48 h, 72 h) establishing the fungal keratitis model, digital photos of the mouse corneas were captured under a slit-lamp microscopy (Fig. 5A).

In the Mock group, there was just slight edema in 6 h and 12 h, and corneal grey-white infiltration occurred after 24 h. In the FK group, the clinical manifestations were similar to the Mock group with scratching and coenobium only in the central cornea at 6 h and 12 h. The central and paracentral cornea had a large area of gray-white infiltration with the iris visible, trauma in central cornea, and hypopyon occurred in the anterior chamber at 24 h. The clinical manifestations continued to worsen at 36 h, with corneal gray-white infiltrated lesions, iris not visible and increasing opacity. While corneal edema aggravated and hypopyon existed at 48 h, the central corneal lesions were reduced. And it showed only central corneal lesions at 72 h, with peripheral corneal transparent and no edema.

In the Block group, the corneal clinical manifestations were similar as that of the FK group and the Mock group at 6 h and 12 h, with no consequential differences in clinical scores (*P* = 1, n = 6 for each time point of the cornea) between the FK group and Block group (Fig. 5A,C). However at 24 h (Fig. 5A): the Block group was characterized as transparent cornea, the iris was clearly visible, with significant differences with FK group in clinical score (Fig. 5A). The differences consisted at 36 h, with no hypopyon formations. Although the Block group manifested corneal opacity at 72 h, it differed from the FK group in lesion characteristics under the slit lamp microscope: restricted dry and dense lesions in the FK group suggested the recovery process of corneal inflammation; however, moist and crisp lesions in the Block group revealed a severe inflammatory reaction. By a total of three batches of experiments (six eyes per group), the clinical score in the Block group was greater than that of the FK group at 72 h (Fig. 5C), and also perforated rates were higher than FK group (*P* = 0.023) (Fig. 5D).

Along with the slit lamp images, histological staining was also performed to understand the histopathology changes underlying the corneal clinical symptom (Fig. 5B). In the FK group, only a small amount of inflammatory cell infiltration were present in the cornea at 6 h and 12 h, and the organizational structure showed no difference from the normal corneas. At 24 h, there was a great deal of inflammatory cell infiltration in the cornea with immune cell underneath the endothelium, as well as increased corneal thickness, and the matrix structure disappeared. Although corneas were damaged, scar tissue within the cornea was formed at 72 h. In the Block group, HE staining showed it underwent no damage in the structure when comparing with the FK group at 6 h and12 h (Fig. 5B), with almost no inflammatory cell infiltration, and it only showed increased corneal thickness without structural destruction at 24 h (Fig. 5B). The differences between the FK group and the Block group were also manifested by clinical scores (*P* < 0.001) (Fig. 5C). After 72 h, the normal corneal morphology disappeared, and all that remained was the organization framework with large number of immune cells adhering to the endothelium.

**Figure 6:**
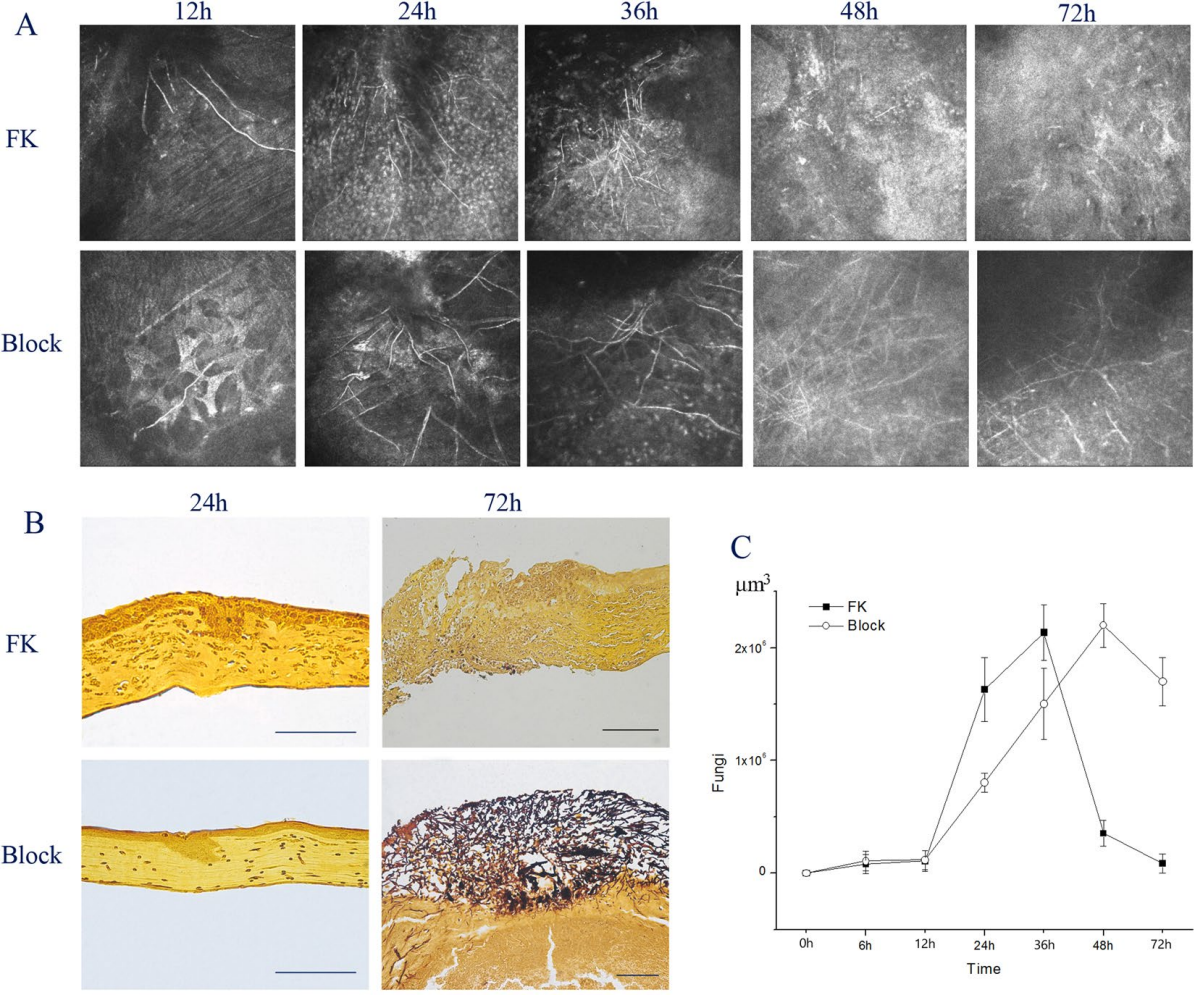
Effects of mast cell activation on corneal histopathology and hyphae. Following photographs of mice corneas under the slit lamp, HRT3 confocal microscopy images (**A**) were screened at 12 h, 24 h, 36 h, 48 h, and 72 h. The hypha was high reflective filament, and the light scatter could be masking it. The GMS staining for FK models was performed (**B**) to observe the fungi growth at different times. (**C**) The volumes of the CFW-positive hypha in the FK group (6 eyes per group, in 4 batches) and the Block group (6 eyes per group, in 4 batches) was measured by the Imaris software. Scale bars for 100 μm (B).

HRT3 confocal microscopy, GMS (Gomori’s methenamine silver) and immunofluorescence staining were performed to examine the growth of fungi. HRT3 images (Fig. 6A) showed that fungus growth was vigorously in 12–36 h, but significantly reduced at 48 h, and there was few fungi detected at 72 h in the FK model. The same results were also showed by GMS staining (Fig. 6B). Fungal volume from immunofluorescence staining (Fig. 6C) was at low level at 6–12 h (*P* = 1.000, *P* = 1.000), increased significantly at 24 h (*P* < 0.001), reached the peak at 36 h (*P* = 0.019), then significantly decreased to low level at 48 h, and very few at 72 h in the FK group. However, the strong growth of fungi continued and lasted from12 to 72 h in the Block group (Fig. 6C), much higher than FK group at 48–72 h, (*P* < 0.001), which suggested that block of blocking MC activation decreased neutrophil suppression of fungal growth in keratitis.

These findings suggest that MC activation by corneal central fungi may cause early neutrophil infiltration from the limbus site and thus lead to severe clinical manifestations in the early stage of fungal keratitis, indirect anti-fungal activity, and finally preventing the late tissue damage caused by fungi growth, decreasing the perforation rate in the late stage.

## Discussion

MCs play a key role in the fight against bacterial infection and viral infection^28,29^, however their role remains poorly understood in the process of corneal fungal infection. This research found that MCs, which are mainly distributed in the corneal limbus, play a dominant role in controlling the fungus infection of the cornea by several mechanisms. This conclusion is based on following evidence: (1) Fungi infection can induce activation and degranulation of MCs in the limbus; (2) cromolyn, the MC stabilizer for degranulation, can significantly prolong the course of disease, increase pathological damage, and increase the perforation rate of fungal keratitis; (3) cromolyn reduces neutrophil infiltration to the site of the fungal infection in the central cornea; (4) MC activation can increase ICAM-1 expression.

Bacteria, parasites, fungi and other physical stimuli can promote MCs activation through the recognition receptors on the cell surface. In the vitro studies, these microbes can directly stimulate the MC degranulation. In this study, the central cornea was irritated by fungi causing MC activation in limbus signaling there are some mechanisms during cell activation. Neurons can secrete nerve growth factors and inflammatory factors, which can bind to the receptors on MC surface following different stimuli^30^. So we conjecture that fungi irritated corneal central nerve fibers to secrete nerve factors and inflammatory factors, then induction of MC degranulation via receptors on MC surface. However, detailed studies are required to establish the definitive mechanism.

Many immune cells participate in fungal infection. There are few evidences on MC in FK. Studies show that MCs can directly kill microorganisms^26^, and also kill bacteria indirectly by chemotaxis of neutrophils^31–33^. MCs can also make direct contact with the bacteria to produce a variety of antibacterial substances, and they can phagocytize and subsequently display the bacterial antigens on their cell surfaces^28,34^. Recent findings have revealed that MCs form the extra-cellular traps which can trap the bacteria to some location to facilitate the anti-bacterial reaction^35,36^. We demonstrated that MCs have an indirect effect on killing fungi in the pathogenesis of FK, via reducing fungal growth by promoting neutrophil infiltration. MC activation is needed for controlling the invasion of fungi was also similar to the study on rat model of acute invasive fungal rhino sinusitis^37^. In a HSV-1 keratitis animal model, research shows that MCs knock-out can reduce the infiltration of neutrophils in the cornea, so as to promote the elimination of virus tilter^18^. In our study, effects of MC activation blocked by cromolyn on neutrophil infiltration is also similar, but the effect on the elimination of fungi is opposite to that of HSV-1, and further studies are needed.

MCs are necessary for corneal wound healing and coordinating neutrophil and platelet recruitment^38^. Eric Pearlman research team confirmed that in the infectious keratitis, corneal haze formation was consistent with neutrophils infiltration^27,39^. The fungal load in neutrophil-knockout mice was higher than the controls^40^, showing that neutrophils are crucial to produce a fungicidal effect. Neutrophils engulf the spores and then kill the fungi or spore through oxygen-dependent or oxygen-independent mechanisms; it can also be formed by extracellular traps to limit fungal hyphae and spore wavering, and then ultimately digest the hyphae or spore through enzymes. In the present study, MCs were located only in the limbus, while the fungal hyphae in the cornea were mainly located in the central cornea. Neutrophils were attracted to the corneal center, where MCs were activated by the fungi. Therefore, it is clear that MCs play an important role in the chemotaxis of neutrophils in fungal keratitis, which have antifungal effects indirectly.

MCs which locate around the blood vessels can promote changes in vascular diameter^41^. Changes of blood vessels were different when MCs activated by different stimuli^42^: After intraperitoneal injection of saline, there were no change in the diameter of the peritoneal blood vessels; after 48/80 stimulation, the vascular diameter decreased at the first 9 days, and then gradually return to normal. The different effect of the two stimuli on vessel diameter may be due to the degree of MC stimulation. Our results suggest that MC activation is less likely to be caused by trauma than by fungal stimulation. This may explain why trauma stimulus does not cause a change in the vessel diameter, whereas a persistent stimulation of the fungus causes increases. Experimental studies showed that 48/80 stimulation of MCs in the early tissue culture (6–8 days) did not increase the vascular permeability of macromolecules, while promotion of vascular permeability of macromolecules occurred in the late stage (9–10 days)^43^. Strbian *et al*. found that cromolyn could restrict hemorrhage formation induced by plasminogen activator^44^. Evans blue can bind with albumin to form Evans blue- albumin complex. In this study, vascular permeability detected by Evans blue concentration was increased by fungal irritation and was higher than trauma irritation but can be suppressed by cromolyn sodium in Block group.

MC activation promotes neutrophil infiltration, blood vessel diameter increases, and ICAM-1 changes. In fungal keratitis, corneal infiltration of inflammatory cells is mainly due to neutrophils. ICAM-1 plays an important role in the chemotaxis of neutrophils during inflammation^45,46^. The infiltration of neutrophils in the blood vessel is divided into three steps: rolling, adhesion, and penetrating. ICAM-1 is crucial for neutrophil amoeboid movement through the vascular wall to the damaged site. We confirmed that the increased expression of ICAM-1 in the cornea initially then decreases in the late stage in the FK group were coherent with the changes in the number of infiltrated neutrophils. The neutrophil infiltration and ICAM-1 expression were blocked by MC inhibitors during the early stage of fungal keratitis.

There are two factors inducing perforation in FK: one is the virulence or mechanical penetration of the fungus itself, and the other is the destruction of the inflammatory cells to the cornea. When neutrophils kill fungi, the intracellular enzyme is released, causing the destruction of corneal tissue^47^. Neutrophil chemotaxis was inhibited in the MC non-activation group, which made it unable to kill fungi. Consequently, the fungal virulence effect is far greater than the destructive effects of neutrophils on the tissue organization, which may be the reason that the perforation rate of the block group was higher than that of the FK group. This demonstrated that MC activation was required for controlling fungal keratitis.

In this experiment, the choice of cromolyn sodium as a MC stabilizer, has certain limitations. Cromolyn sodium can affect macrophages and other inflammatory cells, and it is unclear whether there is an immunosuppressive effect on other cells of the corneal tissue. In addition, studies have shown that in mice, effects of cromolyn sodium on MC membrane stability is lower than that of the other animal *in vivo* effect^48^. Cromolyn may have an effect on the degranulation of other granule cells, such as neutrophils, basophils. To investigate this role, our next step will be to use MC knockout mice to verify this effect. In addition, the role of MCs in mice may be different with that in the human body, and the specific role in the human body still needs further experimental study.

In the present study, we showed connective MCs are found in the corneal limbus and conjunctiva. Corneal fungal infection led to degranulation of these MCs. MC activation can promote vascular changes and increase ICAM-1 expression, thereby promoting neutrophil chemotaxis, as well as affect corneal transparency in the early stage and decrease the perforation rate. The specific reason for MC degranulation caused by fungi infection still needs further investigation. This study adds to evidence on the pathogenesis of fungal keratitis, and may provide information for the treatment of fungal keratitis in the future.

## Materials and Methods

### Animals

All experimental protocols used in these studies were maintained and animals euthanized according to the protocols approved by the Ethical Committee of Experimental Animal Care of Henan Eye Institute. Eight-week-old C57BL/6 J male wild-type mice were purchased from the Model Animal Research Center of Nanjing University (MARC) (Nanjing, China). The mice were maintained on a 12:12-day cycle in a temperature-controlled room at 25 °C.

### Mast cell stabilization and the FK model

Before injection, cromolyn sodium (Sigma-Aldrich, St. Louis, MO) was dissolved in phosphate-buffered saline (PBS, 0.01 M) at a concentration of 4 mg/ml. The cromolyn sodium solution was protected from light during preparation. To stabilize the mast cells, cromolyn sodium (100 mg/kg body weight; Sigma-Aldrich) or vehicle (0.01 M PBS) was administered to the mice by a single intraperitoneal (i.p.) injection for 30–120 min before and after the model^49^, and then additional injections were given every six hours for the duration of the experiment.

The C57BL/6 J mice were randomly divided into three groups, which were the UT group, Mock group, FK group, and Block group, according to the littermate grouping method. The mice were anesthetized by an intraperitoneal injection of sodium pentobarbital (80 mg/kg body weight), and l% (weight/volume) tetracaine hydrochloride eye drops were used for corneal surface anesthesia. Then, under a dissecting microscope, the FK model infected with *Fusarium solani* (F. 3.1791) was built referring to previous methods^50^: using sterile knife to make crossing scratch on the corneal central with the depth of under Bowman’s membrane, then bamboo sticks with fungi hypha scratch 2 to 3 times following the crossing bottom to mimic the natural fungi infection in cornea. Corneas of the Mock group were processed the same procedure above, except fungi infection.

### Corneal clinical score and confocal microscopy

Corneas of three groups were continuously observed and photographed at the corresponding time points under a slit-lamp microscope (SL-8Z, TOPCON, Japan) and a HRT3 confocal microscope (Heidelberg, Germany). Clinical scores were recorded under slit-lamp microscope according to lesion area, depth, corneal neovascularization and others^50^. Parameters of lesion area, depth, corneal neovascularization were given a grade from 0 (normal) to 4 (very severe), and others that contained hypopyon, hyphema, descemetocele and perforation grade from 1 to 4. The fungi hypha and immune cells infiltration were observed by the HRT3 confocal microscope.

### Histologic preparations

For the histologic analysis, mice were euthanized by cervical dislocation at different time points, and the whole cornea and partial sclera were removed and then fixed in 10% neutral formalin (F) for 120 min. Two-micrometer sections were separately stained with Gomori methenamine silver (GMS), or hematoxylin-eosin (HE), and reviewed by light microscopy.

### Observation of Flat-Mounted Cornea

The mice were euthanized by cervical dislocation at different time points. Whole eyes were removed and then fixed with an alcohol-formaldehyde solution (AF) for 120 min. The cornea was trimmed to retain the limbus, but the iris and ciliary body removed. After washing five times with 0.01 M PBS (10 min each), the corneas were dipped in the following solutions in sequence: a 0.2% Triton-BSA solution for 30 min, a 2% BSA solution for 30 min, and the primary antibodies (anti-mouse CD117-FITC provided by Zhijie Li, anti-mouse Tubulin-PE, anti-mouse CD31-PE, anti-mouse MCPT-1, anti-mouse MCPT-6, and anti-mouse Gr-1-FITC, were purchased from eBioscience) incubation for one night. Then, the secondary antibodies (Alexa568, Alexa488, eBioscience) were incubated for 6 hours, washed; incubated in CFW (Sigma-Aldrich, St. Louis, MO) for 30 min, washed; incubated with DAPI for 30 min, and washed. The corneas were then flat mounted with four or eight radial cuts (all of incubation sustained at 4 °C). After multiple immunofluorescence and CFW staining procedures, the flat-mounted corneas of the model groups were photographed by confocal laser scanning microscopy (Z axis, Z = 5 μm). The image analysis was performed by visual inspection of the individual image sections. The different corneal layers were identified by their characteristic morphometric features. Representations of the morphological analyses of the neutrophils and fungal volumes were performed using the Imaris software, version 7.3.1 × 64 (Bitplane, Zurich, Switzerland).

### Vessel diameter and Limbal Vascular permeability

Vessel diameter detection: The Vessel diameter detection was based on the corneal whole-mount technique. Vessel diameter was detected under fluorescent microscope. The limbal microartery in the four equal parts of the cornea was select and captured under 40× objective lens. Then the diameter of limbal microartery was detected on AR software. The limbal microartery is located between two capillary veins. There were four values per cornea, six corneas in every group.

Limbal Vascular permeability: Evans Blue (EB, Sigma, St Louis, MO) in normal saline (2%, 4 ml/kg) was injected intravenously after operation. The mice were killed by cervical dislocation 1 h later and corneas were trimmed. After the tissue was then dried at 100 °C for 24 h, the samples were homogenized in methylformamide (Yongda, Tianjin, China) with 200 μl per cornea, incubated for 24 h at 60 °C, and centrifuged for 5 min at 1000 g. The absorbance (A) of supernatants was analyzed at 632 nm using Envision Multi label plate reader 2104 (Perkin Elmer, USA). The amount of EB (μg/g) was calculated through standard curve established by known concentrations of EB.

### Statistical analysis

The statistical significance of the differences between groups at different times was determined by the one-way analysis of variance test, followed by the least significant difference t (LSD-*t*) test or Kruskal-Wallis Rank Sum test using SPSS V.17.0. A P value of < 0.05 was considered significant. The data are expressed as the means ± STD.

## References

[1] Sauer A (2010). *In vitro* efficacy of antifungal treatment using riboflavin/UV-A (365 nm) combination and amphotericin B. Invest Ophthalmol Vis Sci.

[2] Gopinathan U (2002). The epidemiological features and laboratory results of fungal keratitis: a 10-year review at a referral eye care center in South India. Cornea.

[3] Bharathi MJ, Ramakrishnan R, Vasu S, Meenakshi R, Palaniappan R (2003). Epidemiological characteristics and laboratory diagnosis of fungal keratitis. A three-year study. Indian J Ophthalmol.

[4] Xie L, Zhong W, Shi W, Sun S (2006). Spectrum of fungal keratitis in north China. Ophthalmology.

[5] Bharathi MJ (2006). Microbiological diagnosis of infective keratitis: comparative evaluation of direct microscopy and culture results. Br J Ophthalmol.

[6] Xuguang S, Zhixin W, Zhiqun W, Shiyun L, Ran L (2007). Ocular fungal isolates and antifungal susceptibility in northern China. Am J Ophthalmol.

[7] Gopinathan U, Sharma S, Garg P, Rao GN (2009). Review of epidemiological features, microbiological diagnosis and treatment outcome of microbial keratitis: experience of over a decade. Indian J Ophthalmol.

[8] Wang L (2009). Spectrum of fungal keratitis in central China. Clin Experiment Ophthalmol.

[9] Basak SK, Basak S, Mohanta A, Bhowmick A (2005). Epidemiological and microbiological diagnosis of suppurative keratitis in Gangetic West Bengal, eastern India. Indian J Ophthalmol.

[10] Thomas PA, Kaliamurthy J (2013). Mycotic keratitis: epidemiology, diagnosis and management. Clin Microbiol Infect.

[11] Zhong Jing (2016). Inhibition of trem-1 and dectin-1 alleviates the severity of fungal keratitis by modulating innate immune responses. Plos One.

[12] Branzk N (2014). Neutrophils sense microbe size and selectively release neutrophil extracellular traps in response to large pathogens. Nat immunol.

[13] Sayed BA, Christy AL, Walker ME, Brown MA (2010). Meningeal mast cells affect early T cell central nervous system infiltration and blood-brain barrier integrity through TNF: a role for neutrophil recruitment?. J immunol.

[14] Côté J, Chan H, Brochu G, Chan-Yeung M (1991). Occupational asthma caused by exposure to neurospora in a plywood factory worker. Br J Ind Med.

[15] Vasiadi M (2010). Rupatadine inhibits proinflammatory mediator secretion from human mast cells triggered by different stimuli. Int Arch Allergy Immunol.

[16] Zhang J (2011). Regulation of endothelial cell adhesion molecule expression by mast cells, macrophages, and neutrophils. PLoS One..

[17] Nardo AD, Vitiello A, Gallo RL (2003). Cutting edge: mast cell antimicrobial activity is regulated by casicadin antimicrobial peptide. J Immunol..

[18] Royer DJ, Zheng M, Conrady CD, Carr DJ (2015). Granulocytes in Ocular HSV-1 Infection: Opposing Roles of Mast Cells and Neutrophils. Invest Ophthalmol Vis Sci.

[19] Pinke KH, Lima HG, Cunha FQ, Lara VS (2016). Mast cells phagocyte Candida albicans and produce nitric oxide by mechanisms involving TLR2 and Dectin-1. Immunobiology.

[20] Vardinon N, Segal E, Schwartz J, Eylan E (1975). Mast cell sensitizing antibody (MAST CELLSAb) response in experimental candidiasis: chromatographic studies. Acta Allergologica.

[21] Miller S (1996). Human conjunctival mast cell responses *in vitro* to various secretagogues. Ocular Immunology & Inflammation.

[22] Graziano FM (2001). Conjunctival mast cells in ocular allergic disease. Allergy Asthma Proc.

[23] Liu J (2015). Mast Cells Participate in Corneal Development in Mice. Sci Rep.

[24] Antsiferova M (2013). Mast cells are dispensable for normal and activin-promoted wound healing and skin carcinogenesis. J Immunol.

[25] Farhood A (1995). Intercellular adhesion molecule 1 (ICAM-1) expression and its role in neutrophil-induced ischemia-reperfusion injury in at liver. Journal of Leukocyte Biology.

[26] Yujie G (2015). Platelet-derived Wnt antagonist Dickkopf-1 is implicated in ICAM-1/VCAM-1-mediated neutrophilic acute lung inflammation. Blood.

[27] Taylor PR (2014). Aspergillus and Fusarium corneal infections are regulated by Th17 cells and IL-17-producing neutrophils. J Immunol.

[28] Matsuguchi T (2012). Mast cells as critical effectors of host immune defense against Gram-negative bacteria. Curr Med Chem.

[29] Haidl ID, Marshall JS (2015). Human mast cell activation with viruses and pathogen products. Methods Mol Biol.

[30] Chéret J (2016). 425 Mast cells survival and maturation in human skin are regulated and maintained by sensory nerve fibers. J Invest Dermatol 2016.

[31] Christy AL, Walker ME, Hessner MJ, Brown MA (2013). Mast cell activation and neutrophil recruitment promotes early and robust inflammation in the meninges in EAE. J Autoimmun.

[32] Meuser B (2011). Mast cell function and death in Trypanosoma cruzi infection. Am J Pathol.

[33] Chiba N (2015). Mast cells play an important role in chlamydia pneumoniae lung infection by facilitating immune cell recruitment into the airway. Journal of immunol.

[34] Malaviya R, Navara C, Uckun FM (2002). Augmentation of mast cell bactericidal activity by the anti-leukemic drug, 4-(3′bromo-4′-hydroxylphenyl)-amino-6,7-dimethoxyquinazoline. Leuk Lymphoma.

[35] Branitzki-Heinemann K (2012). A novel role for the transcription factor HIF-1alpha in the formation of mast cell extracellular traps. Biochem J.

[36] Campillonavarro M (2017). Listeria monocytogenes induces mast cell extracellular traps. Immunobiology.

[37] Liu H (2014). Mast cell degranulation in a novel fungus concentration-dependent rat model for acute invasive fungal rhinosinusitis. Pathology.

[38] Burns A, Liu Q, Li Z, Smith C (2013). Mast Cells and the Inflammatory Response to Corneal Epithelial Abrasion. Invest Ophthalmol Vis Sci.

[39] Lee JE (2015). Inhibition of Corneal Inflammation by the Resolvin E1. Invest Ophthalmol Vis Sci.

[40] Leal SM (2012). Fungal antioxidant pathways promote survival against neutrophils during infection. J Clin Invest.

[41] Doyle MP, Linden J, Duling BR (1994). Nucleoside-induced arteriolar constriction: a mast cell-dependent response. Am J Physiol.

[42] Jakobsson AE (1996). Angiogenesis induced by mast cell secretion in rat peritoneal connective tissue is a process of three phases. Microvasc Res.

[43] Rizzo V, DeFouw DO (1996). Mast cell activation accelerates the normal rate of angiogenesis in the chick chorioallantoic membrane. Microvasc Res.

[44] Strbian D, Karjalainen-Lindsberg ML, Kovanen PT, Tatlisumak T, Lindsberg PJ (2007). Mast cell stabilization reduces hemorrhage formation and mortality after administration of thrombolytics in experimental ischemic stroke. Circulation.

[45] Kolaczkowska E, Kubes P (2013). Neutrophil recruitment and function in health and inflammation. Nat rev Immunol.

[46] Gagen D (2010). ICAM-1 mediates surface contact between neutrophils and keratocytes following corneal epithelial abrasion in the mouse. Exp Eye Res.

[47] Wilgus TA, Roy S, McDaniel JC (2013). Neutrophils and Wound Repair: Positive Actions and Negative Reactions. Adv Wound Care.

[48] Oka T, Kalesnikoff J, Starkl P, Tsai M, Galli SJ (2012). Evidence questioning cromolyn’s effectiveness and selectivity as a ‘mast cell stabilizer’ in mice. Lab Invest.

[49] Huang M, Pang X, Karalis K, Theoharides TC (2003). Stress-induced interleukin-6 release in mice is mast cell-dependent and more pronounced in apolipoprotein E knock-out mice. Cardiovasc Res..

[50] Zhang Hongmin (2012). Effects of immunocyte on the process of fungal keratitis. Chin J Exp Ophthalmol.

